# A cross-sectional study reveals complex associations between gastrointestinal parasite composition and faecal immune and inflammatory biomarkers in wildlife and livestock in Kenya

**DOI:** 10.3389/fimmu.2026.1881798

**Published:** 2026-07-28

**Authors:** Alice M. Burton, Andrew M. Halls, Kathryn J. Else, Jessica Irving, Cianjoka Gichuyia, Francis Gakuya, Gabriella K. Davies, Katharine Z. Coyte, Catherine Walton, Samuel M. Githigia, Susanne Shultz

**Affiliations:** 1 Department of Earth and Environmental Science, The University of Manchester, Manchester, United Kingdom; 2 Medical Research Council Centre of Research Excellence in Exposome Immunology (MRC CoRE) for Exposome Immunology, Lydia Becker Institute of Immunology and Inflammation, Division of Immunology, Immunity to Infection and Respiratory Medicine, Faculty of Biology, Medicine & Health, University of Manchester, Manchester, United Kingdom; 3 Wildlife Research and Training Institute, Naivasha, Kenya; 4 Division of Evolution, Infection and Genomics, The University of Manchester, Manchester, United Kingdom; 5 Department of Biology, University of Nairobi, Nairobi, Kenya

**Keywords:** antibodies, ecoimmunology, faecal biomarkers, lactoferrin, livestock, nemabiome, nematodes, wildlife

## Abstract

**Background:**

In landscapes where wildlife and livestock co-graze, hosts are often poly-parasitised with diverse nematode and protozoan communities. The relationships between chronic, endemic parasite infections and host immune response is still unknown in many species, particularly for wildlife. Faecal ecoimmunological biomarkers allow physiological changes to be monitored *in situ*.

**Methods:**

Here we used an exploratory, cross-sectional study to examine the relationships between faecal immunological biomarkers and gastrointestinal parasitism in six wild and four domestic species of ungulates co-grazing in Laikipia, Kenya (10 samples per species). We measured faecal IgA, IgG, bovine lactoferrin and equine IL-6 using in-house enzyme immunoassays, alongside DNA metabarcoding, PCR screening and faecal egg counts, to explore the impacts of parasite composition and load on gut mucosal health. We aimed to identify specific parasites or community characteristic that were associated with higher faecal biomarker concentrations by fitting gobal models and using stepwise selection to identify key parasite predictors.

**Results:**

Interestingly, a comparative immunology approach revealed that no one parasite was associated with a particular biomarker pattern across all host species. For example, the nematode *Cooperia* was associated with a -1.63 fold decrease in IgA and -0.79 decrease in IgG in Bovidae, but with no change in Antelopinae biomarkers. *Nematodirus* was associated with a 1.75-fold increase IgG in giraffe, and with higher IgG (0.65-fold increase) and lactoferrin (1.57-fold increase) in Antelopinae, but was not associated with a change in IgG in Camelidae. *Giardia* was associated with lower IgG in bovids (-0.59-fold decrease), and lower IgA in equids (-0.92-fold decrease) and camels (-0.29-fold decrease). Faecal egg counts were negatively associated with immunoglobulins and inflammatory biomarker concentrations in Antelopinae (-0.23-fold decrease with IgA, -0.39 with IgG and -0.44 with lactoferrin).

**Conclusions:**

This study provides an exploratory overview of the immune responses of wild and domestic herbivores, in association with natural multi-parasitic nematode and protozoan infections. Future research using comparative immunology approaches could improve our understanding of host immune phenotypes and the impacts of co-infections, improving decision making to promote animal health in shared landscapes.

## 1 Introduction

Gastrointestinal parasites are important global infections of both wildlife and livestock, and parasite sharing between related hosts is common (1, 2). In ruminant livestock production systems, nematodes are known to cause significant reductions in animal growth, weight gain, reproduction and productivity (3, 4). In wildlife however, their pathogenicity is often unknown (or is assumed to be negligible), as clinical symptoms are subtle and might only be identified through long-term monitoring or experimental studies (5). Similarly, research into gastrointestinal protozoan parasites is often focused on prevalence and molecular characterisation in humans (6, 7) or livestock (8, 9) rather than wildlife, and the health impacts are often unknown. Despite increasing evidence of parasite community composition provided by DNA barcoding (2, 10, 11), there is still limited evidence for the immunological impacts of chronic infections on their host species, particularly in wildlife, and in environments where animals are highly co-infected and poly-parasitised such as pastoralist livestock systems.

Ecoimmunological biomarkers enable host-pathogen immune and inflammatory relationships to be studied in non-model host species including wildlife and livestock (12–14). Faecal biomarkers in particular offer a useful non-invasive approach, providing specific insights into gastrointestinal health and immune state. Concentrations in faeces represent an aggregation across the gut passage, therefore they may be buffered against short term spikes such as circadian rhythms (although the degree of stability varies between biomarkers). Faecal immunoglobulins can be used as an indicator for investment into immune defences, including both total and parasite-specific antibodies depending on the research question (15–17). IgA is secreted into the gut lumen (as secretory (s) IgA) and is found in high concentrations in excretory products. sIgA maintains mucosal immunity in the gut, through immune exclusion and providing protection against pathogens and toxins (18). Data on the role of IgA in nematode infections in animal hosts is limited, but it is known to be associated with protection of the epithelium in mice and sheep (19). IgG, alongside IgE, is associated with nematode antigen recognition (20, 21). Longitudinal studies of disease ecology in Soay sheep (*Ovis aries*) have used faecal antibodies to identify trade-offs between sub-clinical nematode infections and reproductive output (22), and have found negative correlations between parasite specific antibodies and faecal egg shedding (17). Interpretation of faecal IgG in the context of nematode infections requires caution, as total faecal IgG is not parasite-specific, but it can still provide useful information on overall mucosal immune activity.

Similarly, faecal inflammatory biomarkers are widely used in human medicine to measure gastrointestinal inflammation, including calprotectin and lactoferrin (23, 24). These neutrophilic glycoproteins are released at sites of inflammation in the gut, and sequester metals (calcium and iron respectively), slowing or preventing the growth and reproduction of bacteria and eukaryotic parasites. Within animal research, they can be used to identify gastrointestinal pathology, for example as a result of disease or dietary changes (25, 26). Furthermore, cytokines are key immune signalling molecules that regulate inflammation and immune cell activation. Dysregulation of pro-inflammatory cytokines such as interleukin (IL) – 6 (27) are known to be associated with inflammatory disease states in human patients (28, 29). Faecal pro-inflammatory cytokines have also been used as non-invasive biomarkers of inflammation in animals, such as in dogs with acute diarrhoea (30), and have been used in ecological settings, for example seasonal patterns of parasitism and immune state in semi-feral ponies.

Here we address whether gastrointestinal parasite community composition and infection intensity are associated with biomarkers of gut health in co-grazing wild and domestic ungulates. Laikipia county in central Kenya is a key shared landscape between people, livestock and wildlife, home to semi-nomadic pastoralist communities, and consisting of a mosaic of private and community-operated wildlife conservancies and livestock ranches (31). Historic and ongoing land use changes are resulting in increased pressure on shared resources (32), with high (and increasing) numbers of livestock (33, 34). It remains an important region for biodiversity in Kenya, however; active conservation supports a high diversity of wildlife, including numerous endangered species and large mammals (35). Nematode parasites are known to be shared between wildlife and livestock in Laikipia (1, 2), yet we know little about the immunological or inflammatory responses of natural infections in this landscape. Understanding the immunological interface between wildlife and livestock with natural, chronic, nematode infections is important and supports animal health and One Health aims in this shared ecosystem. For example, livestock health is critical for livelihoods and economic prosperity, therefore awareness of the immune responses to parasites in both livestock themselves and co-grazing wildlife could improve management and disease control decision making.

We explored faecal biomarkers of gastrointestinal immune state and inflammation, specifically IgA, IgG, bovine lactoferrin and equine IL-6, which we have previously demonstrated as relevant to clinical gut pathologies in ruminants (15) and equids. We first investigated relationships between the biomarkers, to identify whether animals had similar immune and inflammatory profiles. Next, we investigated biomarker concentrations in relation to parasite composition using DNA metabarcoding and targeted PCR screening. We predicted that the presence of specific parasites known to be highly pathogenic in livestock will be associated with increased antibody and inflammatory concentrations, in both wildlife and livestock. Finally, we explored two proxies of parasite load, faecal egg shedding and relative read abundance (RRA). We predicted that parasite egg shedding will negatively correlate with faecal immunoglobulin concentrations, and positively with inflammation, and that RRA of tissue-feeding or lumen-associated parasites would be associated with increased inflammatory biomarkers. These markers offer a novel insight into gut health in a highly parasitised assemblage of co-grazing wild and domestic ungulates.

## 2 Materials and methods

### 2.1 Ethics statement

All animal samples used in this project were non-invasive faecal samples. This project was licenced under the National Commission for Science, Technology and Innovation (ref 658799 and 729154), Wildlife Research and Training Institute (WRTI- 0179-04-22 and WRTI-011-11-21), and the Directorate of Veterinary Services for the Ministry of Agriculture and Livestock Development, Kenya (MOALF/SDL/DVS/DS/RESEARCH/VOL I/120). Ethical approval was granted from the University of Manchester Committee for ethical review of category D research (ref D064).

### 2.2 Sample collection

We used a cross-sectional study design, and collected faecal samples during one field season from February – March 2024, from ten herbivore species: cattle (*Bos taurus*), sheep (*Ovis aries*), goats (*Capra aegagrus hircus*), Dromedary camels (*Camelus dromedarius*), African buffalo (*Syncerus caffer*), impala (*Aepyceros melampus*), Reticulated giraffe (*Giraffa reticulata*), African elephant (*Loxodonta africana*), Plains zebra (*Equus quagga*) and Grevy’s zebra (*Equus grevyi*). We observed animals defecating and collected immediately once the animal had moved to a safe distance, ensuring the sample was not contaminated by the ground or other faecal material. We collected a subset of faeces from different parts of the faecal pile to ensure a representative sample and homogenised in a sterile sample bag. We kept samples in a cool box on ice packs until transported to Mpala Research Centre, where they were stored frozen at -20°C (maximum ~4 hours before freezing). Freeze-thaw cycles were kept to a minimum, with 2-3 cycles per sample to enable different analyses. Laikipia typically has a semi-arid savannah shrubland vegetation (36, 37), with around 500 mm rainfall per year.

We included n = 10 samples per species for sequencing of gastrointestinal nemabiomes, selected randomly from adults, with approximately 50/50 male/female ratio. All animals were in good body condition. This number was selected to ensure an even distribution between host species, maximising sequencing depth when multiplexing, and covering a range of host species. Samples were collected oppourtunistically/using convenience sampling on Mpala Research Centre (MRC; 0° 17’ 36.996’’ N, 36° 53’ 54.2256’’ E), a private ranch and research centre, managed for conservation as well as livestock ranching, and has a high density of wildlife in addition to herds of cattle, sheep, and camels. Goats were not being kept on Mpala in 2024, therefore goat samples were collected from Naibung’a Lower Community Conservancy (0° 28’ 37.2072’’ N, 36° 54’ 11.268’’ E), a community conservancy bordering Mpala to the North. Additional giraffe samples were also collected on Naibung’a to supplement the sample size (n = 7 from Mpala, n = 3 from Naibung’a). Finally, African buffalo sightings were extremely rare on Mpala in 2024, therefore n = 10 samples were collected from Loisaba Conservancy (0° 36’ 36.4608” N, 36° 48’ 10.1988” E), a private conservancy bordering Mpala and Naibung’a, with a focus on tourism and conservation but with an active livestock ranch. While we cannot guarantee wildlife samples came from unique individuals, the geographic spread and small sample sizes mean repeat samples are unlikely. Livestock samples were confirmed to be from unique individuals. Livestock on Mpala had previously been treated with anthelmintics (10% Albendazole, 1ml per 10kg body weight). Our final datasets included faecal biomarker concentrations on a total of 159 samples, in addition to sequencing data for parasite prevalence on 127 samples.

### 2.3 Faecal egg shedding and immune biomarkers

We measured gastrointestinal nematode egg shedding using a modified McMaster method as a proxy for parasite burden (38, 39). We homogenised 4g faeces and mixed it with 26 mL saturated sodium chloride (NaCl) solution, poured it through a tea strainer, and pipetted the strained solution into a McMaster slide. After leaving it to stand for 1 minute, we counted the parasite eggs in both chambers at 10X magnification and multiplied the count by 25, giving the final result in eggs per gram of faeces (EPG). We morphologically identified eggs into broad taxonomic groups (strongyles, *Trichuris* spp., and *Moniezia* spp.), in addition to oocysts of coccidia.

We extracted faecal proteins with a solution of protease inhibitor (PI) cocktail (Generon, M5293-1) diluted 1:100 in 1x phosphate-buffered saline (PBS). We added the PI-PBS solution to faecal samples (1:1 – 1:1.5 wet weight: volume depending on consistency and moisture (40, 41)) and homogenised them thoroughly with a toothpick. After leaving the solution to incubate for 20 minutes, we centrifuged them for 5 min at 15,000*g*, and removed the supernatant. We stored the extracts at -20°C before use.

We measured IgA, IgG and lactoferrin using in-house sandwich EIAs, with commercially available anti-bovine coating and detection antibodies (15). We also measured IgA, IgG and IL-6, in zebra using anti-equine antibodies (**Supplementary Materials 1.1**). For camel and giraffe IgA, double the concentration of the coating antibody was used (2 μg/mL) due to low signal. Samples and standards were diluted to their working concentration using 1× PBS containing 0.1% Tween-20 (PBS-T). We ran all samples, standards and controls in duplicate, and calculated coefficients of variance (CVs). We repeated samples with CV > 10%, and excluded any samples with a final CV of >15% after re-runnning. Full assay protocols have been published previously (15), and LODs calculated for IgA; 6.88 ng/mL, IgG; 1.95 ng/mL and lactoferrin; 3.67 ng/mL.

We first analytically validated assays using linearity (linearity (%) = (observed concentration/(previous observed value in the dilution series/dilution Factor))*100) and spike-recovery tests in cattle, and biologically validated them using cattle with diagnosed gastric pathologies (15). We then tested for linearity of dilution in all host species, by serially diluting a pool of faecal extracts for each species and calculating linearity and R^2^ (**Supplementary Table 2.1**). For elephant IgA, and for camel, giraffe and elephant lactoferrin, linearity of dilutions was not satisfied, and signals were very low even when increased concentrations of the coating and detection antibodies were tested. We therefore did not run samples for these assays.

We also tested the stability of the biomarkers in cattle faecal samples (n=10) stored at -80°C, and in extracts stored at -20°C for one year. Concentrations of each biomarker were significantly correlated in samples run a year apart (**Supplementary Materials 3**).

### 2.4 DNA extraction, PCR amplification and sequencing

We extracted DNA from 0.2 g faeces using Zymo Quick-DNA Fecal/Soil Microbe Miniprep Kit (D6010, Zymo Research, California, USA), according to the manufacturer’s protocol. We measured DNA concentration resulting from each extraction using the Qubit^®^ 3.0 Fluorometer (Thermo Fisher Scientific, USA) using the Qubit dsDNA HS Assay Kit (Q32851, Thermo Fisher Scientific, USA).

To identify gastrointestinal nematodes, we used DNA metabarcoding of the ITS-2 and 18S loci of the rRNA cistron using NC1/NC2 (42), which amplify clade V nematodes, and ecoF/ecoR primers (43) for a broader taxonomic range, with Illumina adapters attached (**Supplementary Materials 1.2** for full methods and **Supplementary Materials 4** for primer and index sequences). For bioinformatics we used DADA2 (44) and cutadapt (45), followed by DECIPHER (46) and Kraken2 (47) for assignment (See **Supplementary Materials 1.3** for full methods).

To screen for *Giardia* and *Cryptosporidium*, we used nested PCR to screen for the 18S SSU gene (**Supplementary Materials 1.4**). Reference DNA from either *G. duodenalis* or *Cryptosporidium* spp. provided by the Welsh Cryptosporidium Reference Unit was used as positive controls for reactions.

### 2.5 Statistical analysis

We used R version 4.5.1 for all analyses. We first used principal component analysis (PCA) to investigate whether the three bovine biomarkers loaded together. Not all biomarkers were able to be run for each species due to failure of linearity, therefore we used multiple imputation in the missMDA package (48), first estimating the number of components to use before imputing the missing values. We imputed values in samples where one biomarker was missing; elephants were therefore dropped from the PCA, as neither IgA nor lactoferrin were run. We used a missing at random (MAR) assumption, and imputed values using a regularised iterative PCA approach, which assumes that the data can be represented by a low-dimensional latent structure and that relationships among variables are approximately linear. We scaled and centred concentrartions prior to imputation, and performed imputation using an expectation–maximisation algorithm until convergence. We then calculated a PCA using FactoMineR (49) on scaled biomarker concentrations, and extracted the eigenvalues and loadings for the three biomarkers. A threshold of 1 was used for eigenvalue interpretation. We repeated the PCA calculation without imputing missing values (i.e. dropping camel and giraffe samples, and the overall patterns remained consistent (**Supplementary Figure 2.1**, **Supplementary Table 2.2**).

We converted the nemabiome taxonomy tables into a presence/absence matrix for each parasite genus using tidyverse (50), combining the ITS-2 and 18S datasets, the prevalence of *Giardia* and *Cryptosporidium*, and faecal egg counts. We then grouped data by host family/sub-family (splitting Bovidae into Bovinae (cattle and African buffalo) and Antelopinae (sheep, goats and impala) (51, 52)) and fitted linear models (LMs) using a gaussian distribution and an identity link function, with log10 transformed concentrations as the response, and presence or count of parasites as predictors, per family/sub-family. We performed bidirectional stepwise model selection based on Akaike’s Information Criterion (AIC) using *step* (53). We used this approach (i.e. grouping some host species together) to maximise the use of the species we have, whilst balancing the number of models run. Not all host species could be combined due to low generalisability of the parasite results, for example all elephant parasites were negative in other host species. Combining hosts into one global model could therefore have increased model power/sample size, but would have conflated real vs structural NAs. We visually inspected the residuals of final models for normality, and examined multicollinearity using variance inflation factors (VIFs). We visualised trends between biomarkers and FEC by calculating z scores per biomarker (z = (χ-μ)/σ, where μ = the mean and σ = standard deviation).

We calculated relative read abundance (RRA) of ITS-2 nematodes per sample, as a proxy of parasite infection intensity. We used the ITS-2 dataset for RRA, as the 18S primers also identified a high proportion of non-Nematoda taxa, potentially leading to skews in the relative abundances of nematodes. Finally, we calculated a PCA from the RRA dataset, to investigate if there were patterns between compositional changes and faecal biomarkers. We excluded elephants and equids from this analysis as their genera was not overlapping with other species, and therefore masked differences between samples of other hosts. We extracted PC scores and fitted linear models with log10 transformed biomarker concentrations as the response, and PC1 score with an interaction term with host species as the predictors. We then used *emtrends* in emmeans (54) to test for trends within each species.

## 3 Results

### 3.1 Biomarkers separate between inflammatory and immunoglobulin responses

**Figure 1:**
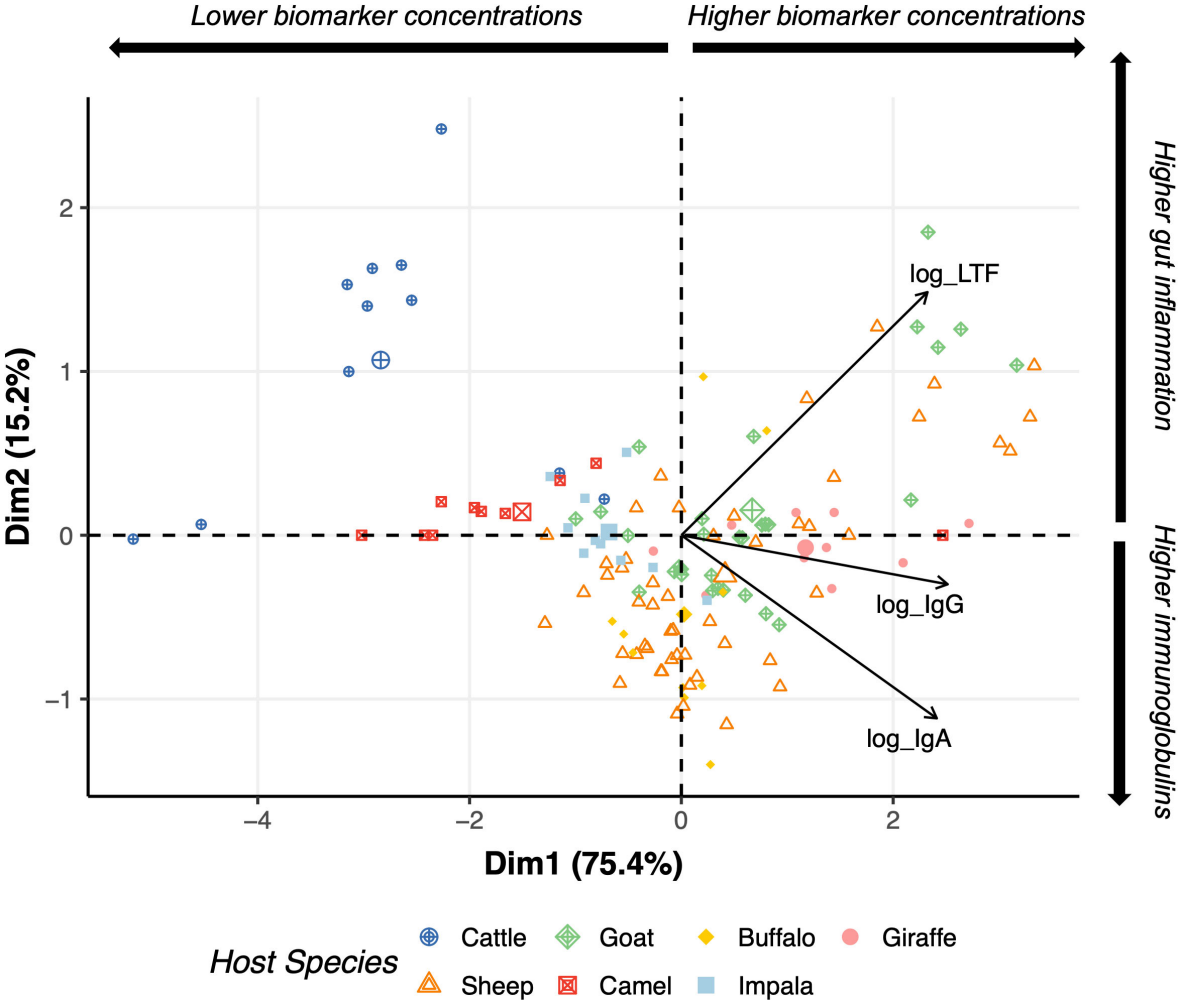
Biomarker PCA of faecal lactoferrin, IgA and IgG in each host species. Species shown are ruminant and pseudoruminants. Concentrations (ng/g) were log10 transformed and normalised for PCA calculation. Dim1 denotes increasing immune biomarker concentrations, whereas Dim2 denotes increasing inflammation. Colour and shape of points denote host species. Livestock are open shapes, wildlife are filled.

In ruminants (and pseudoruminants), concentrations of all three biomarkers loaded together along Dim1 of the biomarker PCA, which denoted increasing concentrations of immune biomarkers in the faeces (**Figure 1**) and explained 75.4% of variation. Biomarker Dim2 represented gastrointestinal inflammation, with lactoferrin loading positively, and the two immunoglobulins negatively. The eigenvalue for biomarker Dim2 was <1 (**Supplementary Table 2.2**), meaning it did not explain more variation than the individual components, therefore lactoferrin concentration was retained for subsequent analysis, rather than Dim2 scores. For consistency, IgA and IgG concentrations were also retained for subsequent analyses, rather than reducing the data into Dim1 scores.

### 3.2 Gastrointestinal parasite composition is associated with faecal biomarkers

**Figure 2:**
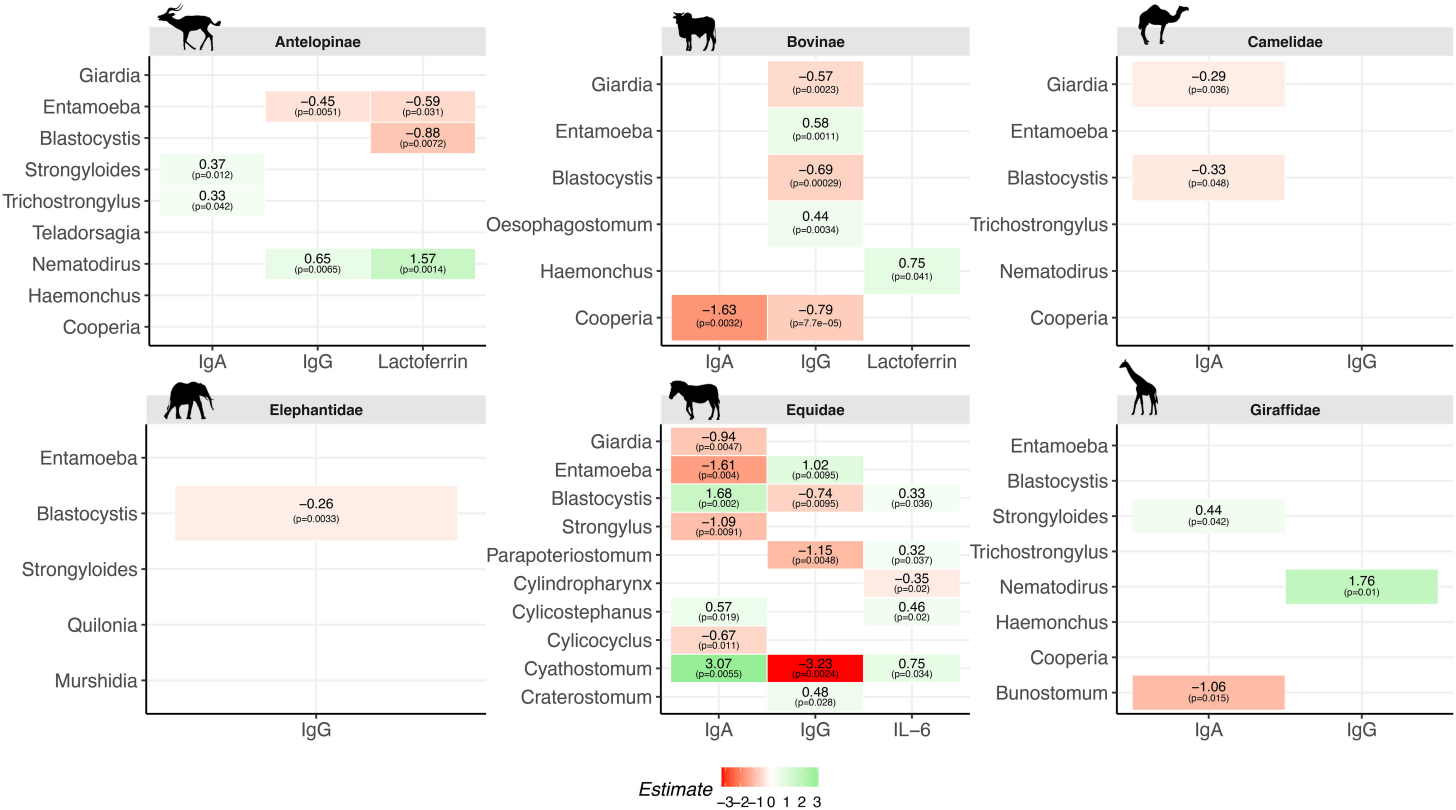
Heatmap showing significant predictors of faecal IgA, IgG and lactoferrin in herbivore species, grouped by family / sub-family. Global models were fit for each family / sub-family, with log10 transformed biomarker concentrations (ng/g faeces) as the response, and the presence/absence of each genus found within the host group as predictors, and bidirectional stepwise model selection was used to select final models. Parasites shown on the y axes represent those identified in samples of each taxon. Statistics shown in each tile are the estimate and *p* value derived from the model output after stepwise selection, i.e. those retained in the final model, with *p* < 0.05 and estimate > 0.2 (or < -0.2 for negative relationships). Full model outpouts including confidence intervals are shown in **Supplementary Table 2.3**. Tiles are coloured according to the estimate; positive estimates are in green, showing the parasite is associated with an increase in biomarker concentration, and negative estimates are in red. Darker green/red denotes a greater positive/negative relationship with biomarker concentration.

Parasite richness was highest in equids (**Supplementary Figure 2.2**), and within the sub-family Antelopinae it was higher in impalas than goats (Tukey HSD: difference = 1.93, *p* = 0.008) and sheep (Tukey HSD: difference = 1.81, *p* = 0.006), whereas there were no differences between cattle and African buffalo within the sub-family Bovinae (Tukey HSD: difference = -0.11, *p* = 0.87). Gastrointestinal parasite community composition was associated with faecal biomarker concentrations differently across the families/sub-families (**Supplementary Table 2.3**, **Figure 2**). For example, we found a decrease in IgA concentrations with the presence of *Giardia* in camels and Equidae, and a decrease with IgG in Bovinae. We found *Entamoeba* was associated with higher IgG in Bovinae and Equidae, but lower IgA in Equidae and lower lactoferrin in Antelopinae. *Blastocystis* was associated with higher IgA and IgG in equids and inflammation in equids (IL-6), but lower IgG in elephants, IgG in Equidae, and lactoferrin in Antilopinae (**Supplementary Table 2.3**, **Figure 2**).

Within nematodes, biomarker concentrations increased with the presence of *Trichostrongylus* (Antelopinae IgA)*, Strongyloides* (Antelopinae IgA, Elephantidae IgG, Giraffidae IgA)*, Oesophagostomum* (Bovinae IgG)*, Nematodirus* (Antelopinae lactoferrin, Giraffidae IgG)*, Cylicostephanus* (Equidae IgA and IL-6 and *Craterostomum* (Equidae IgG), whereas they deceased with the presence of *Cooperia* (Bovinae IgA, Bovinae IgG)*, Bunostomum* (Giraffidae IgA)*, Haemonchus* (Bovinae lactoferrin)*, Cylicocyclus* (Equidae IgA) *Parapoteriostomum* (Equidae IgG e and IL-6) and *Strongylus* (Equiidae IgA) (**Supplementary Table 2.3**, **Figure 2**). In Equidae, we found conflicting relationships with *Cyathostomum*, which was associated with higher IgA but lower IgG) (**Supplementary Table 2.3**, **Figure 2**).

### 3.3 Proxies of parasite infection intensity

**Figure 3:**
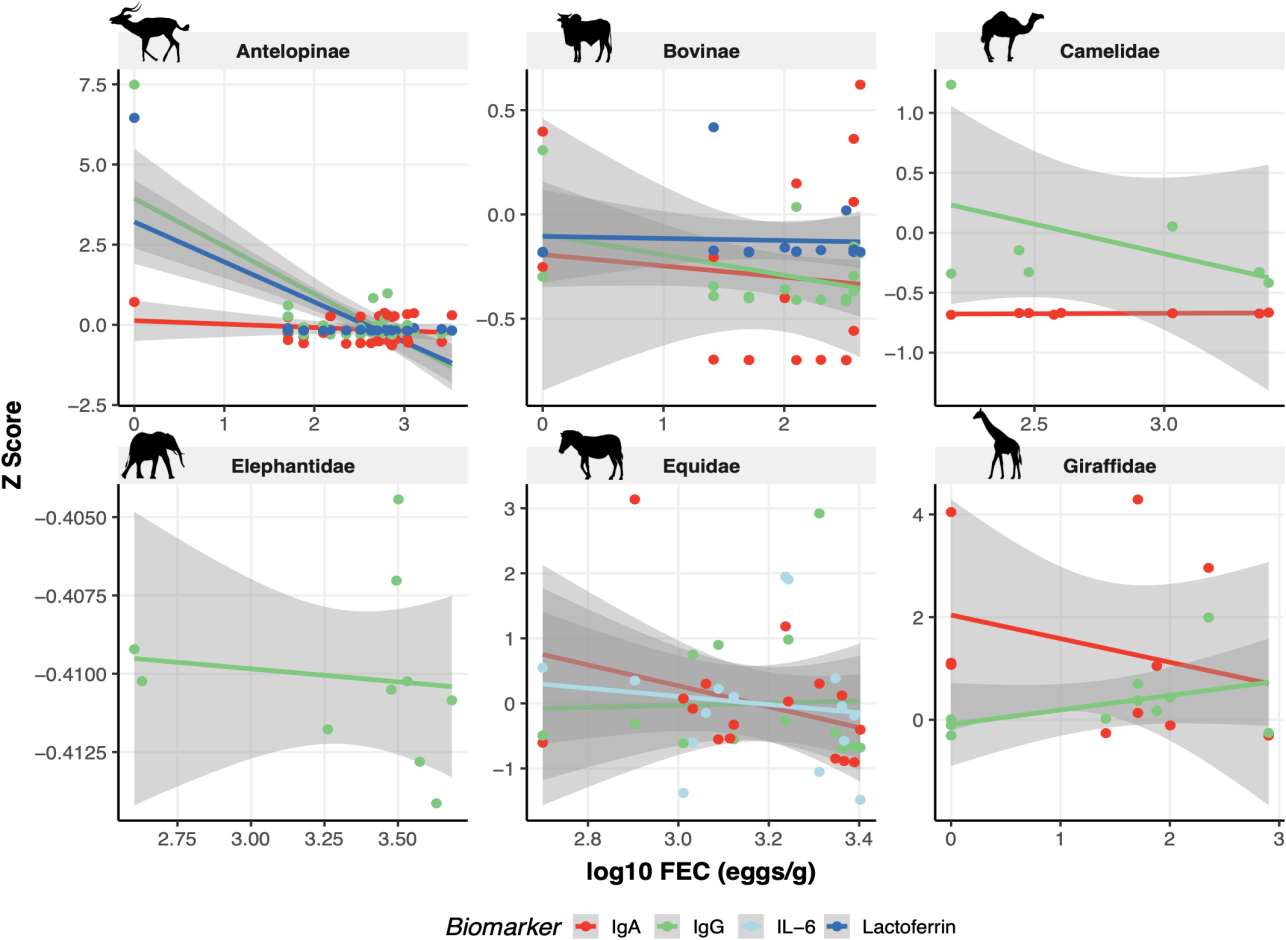
Scatterplot showing relationships between faecal egg count and faecal IgA, IgG and lactoferrin in herbivore species, grouped by family / sub-family. Z scores were calculated from the concentrations (ng/g faeces) of each biomarker. IgA scores are in red, IgG in green, IL-6 in light blue and lactoferrin in dark blue. FEC are log10 transformed. Lines are linear regressions plotted in ggplot2, grey shaded area shows standard error.

We also explored the impact of nematode faecal egg shedding on biomarkers, as a proxy for parasite reproductive output/load. Total FECs were higher in hindgut fermenters (elephants and equids) (**Supplementary Figure 2.2**), but did not vary between host species within Bovinae (Anova: F_1,14_ = 1.23, *p* = 0.27) nor Antelopinae (Anova: F_2,26_ = 0.36, *p* = 0.70). After stepwise selection, FEC was retained in the final models for three sub-families (**Supplementary Table 2.3**). We found negative associations between IgA and IgG with FEC in Antelopinae and Equidae, in addition to negative associations with lactoferrin in Antelopinae, and weak positive associations with IgA and IgG in Bovinae (**Supplementary Table 2.3**, **Figure 3**).

Within the sub-families, host species were associated with differences in biomarker concentrations. Within Antelopinae, goats had higher lactoferrin than sheep and impala, higher IgA than impala, and lower IgG than sheep. In Bovinae, lactoferrin was higher in cattle than African buffalo, whereas IgA and IgG was lower. In Equidae, plains zebra had lower IgG, but higher IgA, than Grevy’s zebra (**Supplementary Table 2.3**).

**Figure 4:**
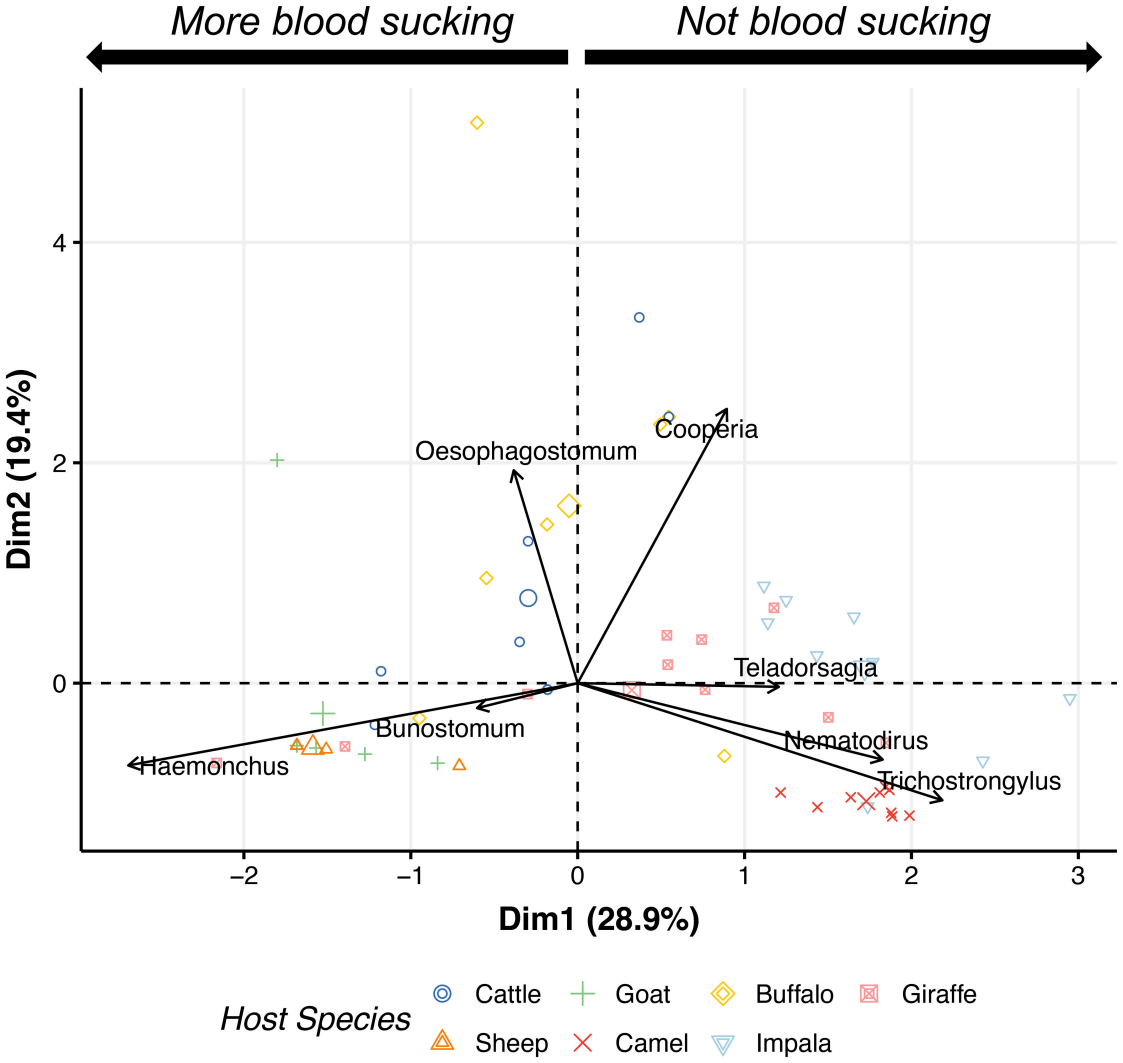
Nemabiome PCA of ruminant (and pseudoruminant) nematode composition, using relative read abundance. Hematophagous parasites (*Haemonchus* and *Bunostomum*) loaded to the left of PC1, whereas non-hematophagous parasites were at the centre or the right. Length of arrows indicate magnitude of loading scores. RRA was calculated from ITS-2 DNA metabarcoding.

Finally, to investigate the impacts of relative compositional changes of the nemabiome on biomarker concentrations, we fitted a PCA of relative read abundance (RRA) of clade V nematodes (hereinafter nemabiome PCA). The RRA of hematophagous (blood feeding) parasites (*Haemonchus* and *Bunostomum*) loaded negatively on nemabiome PCA Dim1, whereas non-hematophagous genera loaded positively (**Figure 4A**, **Supplementary Table 2.5**). Nemabiome PCA Dim2 and Dim3 also had eigenvalues > 1 (**Supplementary Table 2.6**), but these did not appear to be related to parasite characteristics (such as feeding ecology, location within the host, or nodule formation). There was a negative trend between nemabiome Dim1 values with lactoferrin in cattle (**Supplementary Table 2.7**), suggesting cattle with more hematophagous parasites had higher inflammation.

## 4 Discussion

Faecal biomarkers are useful tools to understand host health and immune investment in novel ecological and One Health contexts. They provide a link between parasite composition and host physiology, allowing a deeper understanding of the impacts of infections on animal health, beyond describing prevalence and observing overt clinical symptoms. This is particularly important for livestock and wildlife in shared landscapes, where management decisions might otherwise be based on the presumed relationships between parasitism and immunology/fitness. In this study, we used molecular and ecoimmunological tools to quantify the associations between gastrointestinal parasites and immunological biomarkers in wildlife and livestock in Kenya.

Across both wildlife and livestock ruminant (and pseudoruminant) host species, we found increasing immune biomarker concentrations loaded together on PC1, whereas there was separation between immunoglobulins and inflammatory responses on PC2. These patterns are consistent with those found using a different panel of faecal biomarkers in horses (*Equus caballus*), which also showed separation between pro-inflammatory and pro-immunoglobulin immune states. Mammalian immune responses to chronic parasitic infections may favour persisting antibody responses rather than acute inflammatory responses (55), and this separation between inflammatory and antibody responses was evident in our faecal biomarker profiles.

Cross-species biomarker assays provide valuable information, particularly where commercially available host-specific antibodies are unavailable (56). However, one limitation of this approach is that there could be differences in assay sensitivity and extraction efficiency between species, for example due to differences in antibody affinity to the target antigens, faecal matrix effects (e.g. diet, fiber, moisture content, tannins and inhibitors), rates of protein degradation during field storage, and the use of livestock standards which might not be quantitatively equivalent for wildlife hosts. While it was not possible to conduct biological validations for each species included in this study, we did perform analytical validations for each species, and included host species in models to account for inter-specific variation. The interpretation of our results is focused on relative changes within host species rather than absolute concentrations. Furthermore, the genetic and protein structure of Lactoferrin is highly conserved between hosts, particularly within Bovidae species (57). Any future expansion of these biomarkers into diagnostic contexts will require extensive species-specific biological and analytical validations.

In poly-parasitic environments, coinfection dynamics are complex, and this was evident in our results. For example, in Bovinae, the presence of the nematode *Cooperia*, and the protozoan *Giardia* were associated with a decrease in faecal IgG, whereas *Oesophagostomum* and *Entamoeba* were associated with an increase in IgG. Several studies have reported trade-offs between nematode and protozoan or bacterial pathogens, due to the balance of Th2 vs Th1 type responses needed for each type of infection (58, 59). Even between nematode infections, a study in African buffalo found that clearance of *Haemonchus* induced a Th2 response (which led to increases in coccidia prevalence), whereas clearance of *Cooperia* induced a Th1 response (60). While we cannot make any conclusions about immune state from our total biomarker concentrations, future work could take advantage of parasite-specific antibody assays, in addition to faecal cytokines characterising the Th1/Th2 balance (61), to further explore these trends.

Faecal lactoferrin, which is in an indicator of gut inflammation, was higher in Bovinae with the presence of *Haemonchus*, and higher in Antelopinae with the presence of *Nematodirus.* The presence of *Blastocystis* was associated woth higher pro-inflammatory IL-6 in equids, but with lower lactoferrin in Antelopinae. This was surprising, as protozoans such as *Blastocystis* are known to be associated with inflammatory diseases and diarrhea in livestock (62). Equine IL-6 was also higher with the presence of three nematodes (*Parapoteriostomum, Cylicostephanus* and *Cyathostomum*), but lower with the presence of *Cylindropharynx.* These complex data highlight one of the challenges in studying the ecoimmunology of chronic, endemic parasites in livestock and wildlife, as trends observed from experimental studies or clinical cases might not be replicable in these contexts. Animals with continuous exposure to parasites and high infection and re-infection rates might have very different immune responses compared to those in a more controlled environment (i.e. experimental work). This is becoming evident with laboratory rodent models, as studies of wild house mice (*Mus musculus domesticus*) have revealed context-dependent immune responses to the nematode *Trichuris* that differed from those seen in lab settings (63). This could have practical implications for parasite treatment and control strategies, therefore there is a need to increase the number of comparative immunology studies into livestock and wildlife immunology in the context of chronic poly-parasitism, including with larger sample sizes and expanded suites of biomarkers, to explain these patterns further and evaluate pathology in natural settings. We also acknowledge that due to the sample size constraints, these data represent an exploratory snapshot of the associations between parasites and faecal biomarkers, and other confounding factors such as location, diet, other infectious diseases and livestock management could have contributed to faecal biomarker concentrations. More research is needed to understand ecoimmunology and coinfection in these host species.

We found negative relationships between faecal egg counts and biomarkers in Antelopinae and Equidae, but positive within Bovinae (models accounted for parasite composition, and regressions had wide confidence intervals, so interpretation should be approached with caution). Negative relationships could indicate stronger investment in immune responses leading to lower parasite fecundity (64), although this cannot be concluded from total antibody concentrations. Immune responses to parasite infections are specific, therefore future work using parasite-specific antibodies may improve our understanding of these responses, as has been shown in Soay sheep (17). Furthermore, while FECs provide an easy non-invasive method of estimating parasite load, it should be noted that egg shedding rates do not necessarily represent adult worm burden, due to relative differences in nematode lifecycle stages, fecundity, age of infection, host immunity, season, and nematode species traits. In some host species, FEC does correlate with adult worm burden (65–67), whereas in others this relationship is weaker (68). Despite these caveats, FEC is often used as a proxy for nematode burdens, and we predicted they would be correlated with lower faecal IgG.

Previous work on African equids found positive associations between FEC and faecal IgA in donkeys (*Equus africanus asinus*) in the wet season and with individuals in good body condition, however none in Grevy’s or plains zebra (*Equus grevyi, Equus quagga*), nor donkeys in other seasons or conditions (41). In semi-feral ponies, seasonal patterns in parasitism and immune state have been identified, and faecal pro-inflammatory cytokines peaked in summer, when nematode FECs were also highest, whereas parasite-associated immunoglobulins were highest in late winter/early spring^1^. Our samples were collected during one field season (following a rainy season), and most animals were in good condition, with little variation, therefore future work could explore the impact of seasonal changes on parasite-immune relationships in these species. Since FECs cannot distinguish between taxonomic groups of many gastrointestinal nematodes, for example between genera of strongyle-type eggs, this also highlights the value of molecular methods to identify relationships with specific parasite genera.

When exploring the clade V nematode composition (through a nemabiome PCA based on parasite ITS-2 RRA), we found a separation between the relative proportions of hematophagous (blood sucking) and non-haematophagous (tissue-feeding, mucosa-feeding or lumen-associated) parasites along Dim1. We also found a negative trend between Dim1 and lactoferrin in cattle, suggesting higher inflammation was associated with higher relative abundance of haematophagous parasites. This was somewhat unexpected, as tissue-feeding parasites e.g. *Cooperia* elicit more generalised damage to the mucosa (leading to enteritis in some cases) (69), whereas hematophagous parasites e.g. *Haemonchus* cause direct haemorrhagic damage at feeding sites leading to anaemia (70). Nevertheless, *Haemonchus* are considered to be highly pathogenic in cattle, and these data suggest that shifts in parasite composition, rather than overall burden, can be associated with inflammatory biomarkers. This highlights the importance of thorough diagnostics to identify parasite species in infected animals, especially in poly-parasitised pastoralist landscapes, as therapeutic and preventative treatments should be specific to the infecting species. Lactoferrin can play a role in regulating both Th1 and Th2 responses (71), therefore future studies using faecal cytokines and parasite-specific antibodies could again provide explanations of the underpinning mechanisms driving inflammation in this context.

One limitation of DNA metabarcoding datasets is that they are compositional by nature (72), and cannote be used to measure absolute abundance. There are also inter-nematode biases in extraction efficiency and PCR efficiency, DNA quality, variation in copy numbers of ITS2 genes (even with a species) (73), and differences in egg production rates between nematodes (e.g. *Haemonchus* females can produce thousands of eggs per day (74)). Therefore, while RRA is a useful proxy of composition within samples, it does not represent absolute variation between samples.

This is a first step to understanding the complex roles of gastrointestinal parasites in gut health, immune phenotypes and inflammatory responses in a mixed assemblage of poly-parasitised ungulates in Kenya. In both wildlife and livestock, molecular work and a comparative immunology approach revealed complex associations between nematodes and protozoa with faecal immune and inflammatory biomarkers. Future work using parasite-specific antibodies, in addition to faecal cytokines to quantify the Th1/Th2 response, would improve our understanding of these dynamics. Linking parasite infections to faecal biomarkers can improve our understanding of immunology in natural settings, and of livestock and wildlife health within conservation and One Health contexts.

## Data Availability

The datasets presented in this study can be found in online repositories. The names of the repository/repositories and accession number(s) can be found in the article/**Supplementary Material**.

## Footnotes

1 Irving J, Else KJ, Cruickshank SM, Coyte KZ, Palme R, Shultz S. Inter-individual physiology and gut immune state associates with parasite seasonality in semi-feral, freeranging ponies. (2026).

## Supplementary material

Data Sheet 1 Additional methods.

Data Sheet 2 Additional Tables and Figures.

Data Sheet 3 Biomarker storage stability.

Data Sheet 4 Primer and Index Sequences.

Data Sheet 5 Accession numbers for ITS2 metagenomes.

Data Sheet 6 Accesstion numbers for 18S metagenomes.
