# Supplementary material for: A cross-sectional study reveals complex associations between gastrointestinal parasite composition and faecal immune and inflammatory biomarkers in wildlife and livestock in Kenya
Source: Front Immunol. 2026 Jul 28;17:1881798. doi: 10.3389/fimmu.2026.1881798 (PMC13457056; doi:10.3389/fimmu.2026.1881798)
Supplement: Supplementary file 1 [file DataSheet1.docx]

**Supplementary Materials 1: Additional Methods**

*1.1: Equine assays*

To measure equine IgA, IgG and Il-6, we adapted the protocol previously used for equine and bovine antibodies (1,2). Basic protocols, analytical and biological validations can be found in Irving *et al.,* (3).

*1.2: DNA metabarcoding of nematodes*

For ITS-2 amplification, we used a combination of four forward and four reverse primers each with 0 – 3 N bases between the primer sequence and the Illumina adapter. These heterogeneity spacers aim to increase base diversity during each sequencing cycle, leading to better clustering and fewer sequencing errors. All reactions included a no template control, which included all components of the reaction mixture, but excluded template DNA. PCR reaction mixtures were: 5.2 µL KAPA HiFi HotStart® ReadyMix (Roche, Switzerland), 0.4 µL forward and reverse primers (10 µM), 1 µL template DNA, and 13 µL DNA-free H_2_O. PCR cycling conditions for the ITS-2 amplicon were as follows: 95°C for 1 minute, followed by 40 cycles of 95°C for 15 seconds, 55°C for 20 seconds, and 72°C for 30 seconds, followed by a final extension of 72°C for 5 minutes. For the 18S amplicon, cycling conditions were as follows: 95°C for 3 minutes, followed by 35 cycles of 98°C for 20 seconds, 64°C for 15 seconds, and 72°C for 30 seconds, followed by a final extension of 72°C for 2 minutes. We confirmed amplification using a 1% Agarose Gel, stained with SafeView^TM^ Classic (Applied Biological Materials Inc. (amb), Canada), and visualised under a SmartBlue™ Blue Light Transilluminator (Accuris, Colorado, USA).

We cleaned PCR Products with AppMag PCR Clean Up Beads (Appleton Woods, UK), at 1.8X. We incubated the solution at room temperature for 10 minutes on an orbital shaker, before transferring to a magnetic particle concentrator. Once the solution cleared (2 minutes), we removed the supernatant and washed the beads twice with 20 µl freshly prepared 80% ethanol. We briefly dried the beads, ensuring they do not over-dry and crack, before removing them from the magnet and resuspending in 20 µl warmed nuclease-free water. We incubated the resuspended beads for 10 minutes at room temperature on an orbital shaker, before separating on the magnet for a final 2-minute incubation. We finally removed the clear supernatant and stored at 4°C before proceeding to the next step.

To allow for multiplexing, we carried out a secondary PCR to add dual indexes to each sample, using a unique combination of i5 and i7 indices (Supplementary Material 5.3). We conducted indexing PCRs with 0.1 μl GoTaq® G2 DNA Polymerase (Promega, USA), 5 μl GoTaq G2 colourless reaction buffer, 2.5 μl MgCl_2_ (25 µM), 0.5 μl dNTPs (10mM), 13.375 μl nuclease-free H_2_O, 0.75 μl each of the i5 and i7 index (10 µM), and 2 μl cleaned PCR product. The cycling conditions were: initial denaturing for 95°C for 2 minutes, followed by 15 cycles of 98°C for 20 seconds, 63°C for 20 seconds and 72°C for 2 minutes, and a final extension of 72°C for 2 minutes. We confirmed that indexes had successfully attached using a 1% Agarose Gel. We then bead-cleaned cleaned the indexed PCR products, as above.

We measured DNA concentrations using the Qubit® 3.0 Fluorometer (Thermo Fisher Scientific, USA) using the Qubit dsDNA HS Assay Kit (Q32851, Thermo Fisher Scientific, USA), and normalised each sample to 4nM, before pooling the 4nM libraries (2 μl of each). We denatured the 4nM pooled library and 10nM PhiX with 0.2N NaOH, then diluted to 20pM with HT1 Hybridisation Buffer (Illumina, UK). We prepared a final library at 8pM with a 15% PhiX spike. Libraries were sequenced on a MiSeq System (Illumina, Cambridge, UK) using a V2 500 cycle reagent cartridge, at the International Livestock Research Institute (ILRI), Nairobi, Kenya, with assistance from the ILRI Genomics Team.

*1.3. Bioinformatics*

Bioinformatics and data analysis was conducted in R (R Core Team, 2023), and amplicon sequences were processed using DADA2 (Callahan et al., 2016). Sequencing generated 52,435,552 raw reads in 143 samples for the ITS-2 amplicon, and 45,393,108 reads in 142 samples for the 18S amplicon. We first used cutadapt version 2.6 (Martin, 2011) to identify and remove primer sequences, using default parameters (error rate= 0.1, allowing mismatches and indels, minimum overlap length = 3). We then visually inspected quality profiles in dada2 with plotQualityProfile, and filtered and trimmed them using *filterAndTrim* (maxEE = c(2, 5), truncQ = 2, rm.phix = TRUE). We learned error rates using *learnErrors*, denoised with *dada* and merged reads using *mergePairs* (maxMismatch = 1), before generating a sequence table with *makeSequenceTable* and removed chimeras with *removeBimeraDenovo* (method = consensus).

For ITS-2 taxonomic assignment, we used the DECIPHER package (version 3.2.0) (4), and amplicon-specific nematode reference databases (5,6); we trained the classifier using a training set with *LearnTaxa*, then assigned taxonomy using *IdTaxa* (threshold = 60, bootstraps = 60. The sequence table, taxonomy table, and sample metadata were combined using *phyloseq* (7). For the 18S reads, we used kraken2 (8), with the nt database on paired-end reads using a 0.2 confidence threshold. Kraken2 assignment was conducted on the University of Manchester’s Computational Shared Facility (CSF) using Bourne Again Shell (BASH) scripts and Simple Linux Utility for Resource Management (SLURM) job scheduler. We visualised the output using *ktImportTaxonomy* in krona (9), created a BIOM-format table using *kraken-biom* (10), and imported the BIOM into R using phyloseq. We removed any ASVs present at < 2% of the reads per sample, any ASVs present in blank samples, and those not identified to the Nematoda phylum. We removed blank samples, in addition to eight samples from the ITS-2 dataset with remaining contamination (ruminant species containing equid parasites), and four samples with a high proportion of unassigned reads. We used a centred log ratio (CLR) transformation from the microbiome package (11). The final datasets for ITS-2 contained 288 ASVs in 126 samples, and for 18S contained 323 ASVs in 135 samples.

*1.4: PCR screening of Giardia and Cryptosporidium*

For all the following PCRs we used GoTaq G2 Hotstart Taq Polymerase (M7405, Promega, Madison, WI, USA) in 25 µl reaction volumes consisting of 1x Gotaq G2 hotstart buffer, 2.5mM MgCl_2_, 0.2mM dNTP, forward and reverse primers each at 0.6 µM, and 0.625 U *Taq* polymerase.

For *Giardia,* we used the forward primer RH11 (5′-CATCCGGTCGATCCTGCC-3′) and the reverse primer RH4 (5′-AGTCGAACCCTGATTCTCCGCCAGG-3′) for the primary reaction, amplifying a 292 bp region. We then amplified a 130 bp product using the forward primer GIarF (5′-GACGCTCTCCCCAAGGAC-3′) and the reverse primer GiarR (5′- CTGCGTCACGCTGCTCG -3′) for the nested reaction. The cycling conditions for both steps were: initial denaturing at 96 ^o^C for 4mins, 35 cycles of denaturing at 96 ^o^C for 20s, annealing at 59 ^o^C for 20s and an extension at 72 ^o^C for 30s, followed by a final extension of 7mins at 72 ^o^C (18).

For *Cryptosporidium*, we used primers and annealing temperatures described by Xiao et al. (12). We used the forward primer 5′-TTCTAGAGCTAATACATGCG-3′ and the reverse primer 5′- CCCTAATCCTTCGAAACAGGA-3′ for the primary step, amplifying a product ~1,325 bp. For the nested step we used the forward primer 5′- GGAAGGGTTGTATTTATTAGATAAAG-3 and the reverse primer 5′- AAGGAGTAAGGAACAACCTCCA-3′ to amplify an 826-864bp product. Both of these reactions were carried out under the same conditions: initial denaturing of 94^o^C for 3mins, 35 cycles of denaturing at 94 ^o^C for 45s, annealing at 56 ^o^C for 45s and an extension step at 72 ^o^C for 60s, followed by a final extension step at 72 ^o^C for 7mins.

*1.5: Supplementary References*

1.	Burton AM, Else KJ, Irving J, Mair I, Shultz S. Antibodies and Inflammation: Fecal Biomarkers of Gut Health in Domestic Ruminants. *J Exp Zool Pt A* (2025)jez.2896. doi: 10.1002/jez.2896

2.	Tombak KJ, Budischak SA, Hauck S, Martinez LA, Rubenstein DI. The non-invasive measurement of faecal immunoglobulin in African equids. *International Journal for Parasitology: Parasites and Wildlife* (2020) 12:105–112. doi: 10.1016/j.ijppaw.2020.05.005

3.	Irving J, Else KJ, Cruickshank SM, Coyte KZ, Palme R, Shultz S. Inter-individual physiology and gut immune state associates with parasite seasonality in semi-feral, free-ranging ponies. *[Manuscript submitted for publication]* (2026)

4.	Wright ES. Using DECIPHER v2.0 to Analyze Big Biological Sequence Data in R. *The R Journal* (2016) 8:352–259.

5.	Charrier E, Chen R, Thundathil N, Gilleard JS. A set of nematode rRNA cistron databases and a primer assessment tool to enable more flexible and comprehensive metabarcoding. *Molecular Ecology Resources* (2024) 24:e13965. doi: 10.1111/1755-0998.13965

6.	Workentine ML, Chen R, Zhu S, Gavriliuc S, Shaw N, Rijke J de, Redman EM, Avramenko RW, Wit J, Poissant J, et al. A database for ITS2 sequences from nematodes. *BMC Genet* (2020) 21:74. doi: 10.1186/s12863-020-00880-0

7.	McMurdie PJ, Holmes S. phyloseq: An R Package for Reproducible Interactive Analysis and Graphics of Microbiome Census Data. *PLoS ONE* (2013) 8:e61217. doi: 10.1371/journal.pone.0061217

8.	Wood DE, Lu J, Langmead B. Improved metagenomic analysis with Kraken 2. *Genome Biology* (2019) 20:257. doi: 10.1186/s13059-019-1891-0

9.	Ondov BD, Bergman NH, Phillippy AM. Interactive metagenomic visualization in a Web browser. *BMC Bioinformatics* (2011) 12:385. doi: 10.1186/1471-2105-12-385

10.	Dabdoub SM. kraken-biom: Enabling interoperative format conversion for Kraken results. (2016) https://github.com/smdabdoub/kraken-biom.

11.	Lahti L, Shetty S. microbiome. (2017) doi: 10.18129/B9.BIOC.MICROBIOME
