# Supplementary material for: A cross-sectional study reveals complex associations between gastrointestinal parasite composition and faecal immune and inflammatory biomarkers in wildlife and livestock in Kenya
Source: Front Immunol. 2026 Jul 28;17:1881798. doi: 10.3389/fimmu.2026.1881798 (PMC13457056; doi:10.3389/fimmu.2026.1881798)
Supplement: Supplementary file 2 [file DataSheet2.docx]

**Supplementary Materials 2: Additional Tables and Figures**

**Supplementary Table 2.1: Linearity of dilutions and R2 of pooled faecal extracts for each species.** Two-fold serial dilutions of extracts pooled from individuals of each species.

|  | IgA | | IgG | | Inflammation (Lactoferrin / IL-6)^§^ | |
| --- | --- | --- | --- | --- | --- | --- |
|  | R^2^ | Linearity (%) | R^2^ | Linearity (%) | R^2^ | Linearity (%) |
| Cattle | 0.97 | 108.46 | 0.99 | 117.65 | 0.99 | 111.11 |
| Sheep | 0.93 | 106.23 | 0.93 | 121.82 | 0.93 | 81.03 |
| Goat | 0.93 | 110.19 | 0.99 | 108.24 | 0.98 | 119.73 |
| Camel | 0.99 | 130.11 | 1.00 | 121.98 | 0.87 | 138.13^†^ |
| Buffalo | 0.86 | 108.55 | 0.97 | 129.54 | 0.98 | 95.97 |
| Impala | 0.95 | 104.52 | 0.88 | 122.27 | 0.99 | 123.32 |
| Giraffe | 0.97 | 107.76 | 0.98 | 116.79 | 0.81 | 188.63^†^ |
| Elephant | 0.65 | 138.01^†^ | 0.98 | 121.75 | 0.73 | 207.65^†^ |
| Plains zebra^‡^ | 0.97 | 108.73 | 0.99 | 110.85 | 0.97 | 154.95 |
| Grevy’s zebra^‡^ | 0.97 | 111.62 | 0.99 | 115.41 |  |  |

^†^IgA was not linear for elephants, and lactoferrin was not linear for camels, giraffe, or elephants. Signals were low for these species indicating low concentrations or poor cross-reactivity of bovine antibodies.

^‡^Anti-horse antibodies

^§^For plains and Grevy’s zebra Il-6 was used as an inflammatory biomarker. For other species, anti-bovine lactoferrin was used. Grevy’s and plains zebra were combined in one zebra pool to assess Il-6 linearity.

Biomarkers loaded together on PC1, which had an eigenvalue >1. Lactoferrin was separated from the immunoglobulins on PC2 (Supplementary Table 2.2).

**Supplementary Table 2.2: Eigenvalues and loading scores for PCA of biomarker concentrations with multiple imputation.**

These patterns were consistent without multiple imputation of missing values (Supplementary Figure 2.1). Camels and giraffe were dropped as they did not have lactoferrin values. PC1 explained slightly less variation without imputing missing values, and PC2 explained slightly more (Supplementary Table 2.3).

**Supplementary Table 2.3: Parasite composition predicts faecal biomarker concentrations.** Results following stepwise selection on global models with the prevalence or count (FEC only) of parasite genera that were identified in samples within each sub-family as predictors, and biomarker concentration (log10 transformed IgA, IgG or lactoferrin) as responses.

|  | IgA | | | | | IgG | | | | | Lactoferrin | | | | |
| --- | --- | --- | --- | --- | --- | --- | --- | --- | --- | --- | --- | --- | --- | --- | --- |
|  | term | estimate | std.error | statistic | *p* | term | estimate | std.error | statistic | *p* | term | estimate | std.error | statistic | *p* |
| Antelopinae | Teladorsagia | -0.262 | 0.164 | -1.601 | 0.119 | **Nematodirus** | **0.646** | **0.222** | **2.908** | **0.006** | **Nematodirus** | **1.57** | **0.449** | **3.498** | **0.001** |
|  | **Trichostrongylus** | **0.331** | **0.157** | **2.113** | **0.042** | **Trichostrongylus** | **0.331** | **0.177** | **1.865** | **0.071** | **Blastocystis** | **-0.876** | **0.306** | **-2.863** | **0.007** |
|  | **Strongyloides** | **0.371** | **0.14** | **2.648** | **0.012** | Strongyloides | 0.202 | 0.15 | 1.344 | 0.188 | **Entamoeba** | **-0.586** | **0.26** | **-2.253** | **0.031** |
|  | Entamoeba | -0.236 | 0.133 | -1.771 | 0.085 | **Entamoeba** | **-0.451** | **0.15** | **-3.004** | **0.005** | Trichuris.epg | -0.004 | 0.002 | -2.013 | 0.052 |
|  | **log10_epg** | **-0.228** | **0.083** | **-2.755** | **0.009** | Giardia | 0.197 | 0.14 | 1.408 | 0.168 | Coccidia.opg | 0 | 0 | 1.808 | 0.08 |
|  | **HostImpala** | **-0.888** | **0.18** | **-4.929** | **<0.001** | **log10_epg** | **-0.393** | **0.09** | **-4.351** | **<0.001** | **log10_epg** | **-0.436** | **0.169** | **-2.59** | **0.014** |
|  | HostSheep | -0.08 | 0.166 | -0.482 | 0.633 | HostImpala | -0.475 | 0.283 | -1.681 | 0.102 | **HostImpala** | **-1.973** | **0.524** | **-3.765** | **0.001** |
|  |  |  |  |  |  | **HostSheep** | **0.554** | **0.181** | **3.065** | **0.004** | **HostSheep** | **-0.83** | **0.326** | **-2.544** | **0.016** |
| Bovinae | **Cooperia** | **-1.632** | **0.344** | **-4.748** | **0.003** | **Cooperia** | **-0.79** | **0.083** | **-9.516** | **<0.001** | Cooperia | -0.662 | 0.363 | -1.822 | 0.111 |
|  | Oesophagostomum | 0.549 | 0.337 | 1.63 | 0.154 | **Blastocystis** | **-0.691** | **0.092** | **-7.526** | **<0.001** | **Haemonchus** | **0.754** | **0.301** | **2.505** | **0.041** |
|  | Blastocystis | -0.376 | 0.327 | -1.149 | 0.294 | **Oesophagostomum** | **0.443** | **0.094** | **4.692** | **0.003** | Oesophagostomum | 0.629 | 0.364 | 1.727 | 0.128 |
|  | Entamoeba | 0.717 | 0.38 | 1.889 | 0.108 | **Entamoeba** | **0.582** | **0.1** | **5.806** | **0.001** | Blastocystis | 0.445 | 0.222 | 2.001 | 0.086 |
|  | Giardia | -0.48 | 0.354 | -1.357 | 0.224 | **Giardia** | **-0.569** | **0.112** | **-5.068** | **0.002** | Strongyles.epg | 0.003 | 0.001 | 2.102 | 0.074 |
|  | Coccidia.opg | 0.011 | 0.009 | 1.315 | 0.236 | **Trichuris.epg** | **0.001** | **0** | **2.676** | **0.037** | **Coccidia.opg** | **0.055** | **0.011** | **4.853** | **0.002** |
|  | **log10_epg** | **0.813** | **0.215** | **3.781** | **0.009** | **log10_epg** | **0.36** | **0.053** | **6.837** | **<0.001** | **HostCattle** | **1.461** | **0.417** | **3.505** | **0.01** |
|  | **HostCattle** | **-2.115** | **0.193** | **-10.933** | **<0.001** | **HostCattle** | **-1.158** | **0.073** | **-15.763** | **<0.001** |  |  |  |  |  |
| Camelidae | Cooperia | 0.083 | 0.041 | 2.036 | 0.179 | Nematodirus | 1.188 | 0.73 | 1.628 | 0.202 |  |  |  |  |  |
|  | Nematodirus | 0.191 | 0.054 | 3.534 | 0.072 | Blastocystis | 0.772 | 0.665 | 1.16 | 0.33 |  |  |  |  |  |
|  | **Blastocystis** | **-0.335** | **0.076** | **-4.377** | **0.048** | log10(Strongyles.epg + 1) | -1.216 | 0.522 | -2.33 | 0.102 |  |  |  |  |  |
|  | **Giardia** | **-0.287** | **0.056** | **-5.097** | **0.036** |  |  |  |  |  |  |  |  |  |  |
|  | **log10(Strongyles.epg + 1)** | **0.329** | **0.067** | **4.883** | **0.039** |  |  |  |  |  |  |  |  |  |  |
| Elephantidae |  |  |  |  |  | **Blastocystis** | **-0.264** | **0.05** | **-5.272** | **0.003** |  |  |  |  |  |
|  |  |  |  |  |  | Entamoeba | 0.072 | 0.032 | 2.228 | 0.076 |  |  |  |  |  |
|  |  |  |  |  |  | **Strongyloides** | **0.113** | **0.03** | **3.751** | **0.013** |  |  |  |  |  |
|  |  |  |  |  |  | **Strongyles.epg** | **0** | **0** | **-2.96** | **0.032** |  |  |  |  |  |
| Equidae | Craterostomum | 0.179 | 0.052 | 3.459 | 0.074 | **Craterostomum** | **0.482** | **0.157** | **3.076** | **0.028** |  |  |  |  |  |
|  | **Cyathostomum** | **3.068** | **0.228** | **13.481** | **0.005** | **Cyathostomum** | **-3.23** | **0.571** | **-5.652** | **0.002** |  |  |  |  |  |
|  | **Cylicocyclus** | **-0.673** | **0.073** | **-9.267** | **0.011** | **Cylicocyclus** | **-0.242** | **0.18** | **-1.347** | **0.236** |  |  |  |  |  |
|  | **Cylicostephanus** | **0.567** | **0.079** | **7.208** | **0.019** | **Cylindropharynx** | **0.527** | **0.21** | **2.511** | **0.054** |  |  |  |  |  |
|  | Cylindropharynx | -0.163 | 0.072 | -2.255 | 0.153 | **Parapoteriostomum** | **-1.153** | **0.239** | **-4.819** | **0.005** |  |  |  |  |  |
|  | Parapoteriostomum | -0.126 | 0.084 | -1.49 | 0.275 | Strongylus | 0.8 | 0.313 | 2.554 | 0.051 |  |  |  |  |  |
|  | **Strongylus** | **-1.086** | **0.105** | **-10.392** | **0.009** | **Blastocystis** | **-0.738** | **0.181** | **-4.084** | **0.01** |  |  |  |  |  |
|  | **Blastocystis** | **1.681** | **0.076** | **22.263** | **0.002** | **Entamoeba** | **1.015** | **0.248** | **4.089** | **0.009** |  |  |  |  |  |
|  | **Entamoeba** | **-1.614** | **0.102** | **-15.834** | **0.004** | **HostPlains zebra** | **-1.354** | **0.172** | **-7.888** | **0.001** |  |  |  |  |  |
|  | **Giardia** | **-0.942** | **0.065** | **-14.495** | **0.005** |  |  |  |  |  |  |  |  |  |  |
|  | **Strongyles.epg** | **-0.001** | **0** | **-16.086** | **0.004** |  |  |  |  |  |  |  |  |  |  |
|  | **HostPlains zebra** | **0.655** | **0.081** | **8.112** | **0.015** |  |  |  |  |  |  |  |  |  |  |
| Giraffidae | **Bunostomum** | **-1.065** | **0.212** | **-5.014** | **0.015** | Cooperia | -1.149 | 0.39 | -2.946 | 0.06 |  |  |  |  |  |
|  | Cooperia | -0.998 | 0.463 | -2.157 | 0.12 | Haemonchus | 0.159 | 0.131 | 1.209 | 0.313 |  |  |  |  |  |
|  | Nematodirus | 0.998 | 0.38 | 2.627 | 0.079 | **Nematodirus** | **1.756** | **0.3** | **5.844** | **0.01** |  |  |  |  |  |
|  | Trichostrongylus | -0.366 | 0.175 | -2.088 | 0.128 | Trichostrongylus | 0.122 | 0.097 | 1.253 | 0.299 |  |  |  |  |  |
|  | **Strongyloides** | **0.438** | **0.128** | **3.413** | **0.042** | Strongyloides | -0.103 | 0.114 | -0.9 | 0.434 |  |  |  |  |  |
|  | **Strongyles.epg** | **0.004** | **0.001** | **3.689** | **0.035** | **Strongyles.epg** | **0.003** | **0.001** | **4.887** | **0.016** |  |  |  |  |  |

Supplementary Figure 2.1: PCA of faecal lactoferrin, IgA and IgG in each host species, without multiple imputation of missing values. Concentrations (ng/g) were log10 transformed and normalised for PCA calculation. PC1 denotes increasing immune biomarker concentrations, whereas PC2 denotes increasing inflammation. Colour and shape of points denote host species.

**Supplementary Table 2.4: Eigenvalues and loading scores for PCA of biomarker concentrations without multiple imputation.**

Supplementary Figure 2.2 Variation in total FEC (A) and parasite richness (B) between host species. Parasite richness is presented as the total number of parasites present in two or more samples per host species.

**Supplementary Table 2.5: Loading scores for each genus on the nemabiome PCA**

**Supplementary Table 2.6: Eigenvalues of the nemabiome RRA PCA**

**Supplementary Table 2.7: Estimated marginal means of linear trends comparing the relationships between nemabiome PCA Dim1 scores and biomarker concentrations.**

|  | Host | Trend | SE | df | lower.CL | upper.CL | t.ratio | *p* |
| --- | --- | --- | --- | --- | --- | --- | --- | --- |
| IgA | Buffalo | -0.057 | 0.233 | 48 | -0.526 | 0.411 | -0.246 | 0.807 |
|  | Camel | 0.387 | 0.616 | 48 | -0.853 | 1.626 | 0.627 | 0.533 |
|  | Cattle | 0.113 | 0.236 | 48 | -0.363 | 0.587 | 0.476 | 0.636 |
|  | Giraffe | 0.14 | 0.104 | 48 | -0.07 | 0.349 | 1.336 | 0.188 |
|  | Goat | 0.131 | 0.471 | 48 | -0.816 | 1.077 | 0.277 | 0.783 |
|  | Impala | -0.052 | 0.228 | 48 | -0.51 | 0.406 | -0.228 | 0.821 |
|  | Sheep | -0.31 | 0.428 | 48 | -1.17 | 0.55 | -0.725 | 0.472 |
| IgG | Buffalo | -0.265 | 0.248 | 47 | -0.763 | 0.233 | -1.07 | 0.29 |
|  | Camel | -0.715 | 0.61 | 47 | -1.942 | 0.512 | -1.173 | 0.247 |
|  | Cattle | 0.144 | 0.251 | 47 | -0.361 | 0.648 | 0.572 | 0.57 |
|  | Giraffe | 0.034 | 0.111 | 47 | -0.189 | 0.258 | 0.311 | 0.758 |
|  | Goat | -0.362 | 0.501 | 47 | -1.369 | 0.645 | -0.722 | 0.474 |
|  | Impala | 0.129 | 0.242 | 47 | -0.358 | 0.616 | 0.533 | 0.597 |
|  | Sheep | -0.035 | 0.452 | 47 | -0.944 | 0.874 | -0.077 | 0.939 |
| Lactoferrin | Buffalo | -0.218 | 0.404 | 34 | -1.039 | 0.603 | -0.54 | 0.593 |
|  | Cattle | -0.722 | 0.41 | 34 | -1.554 | 0.11 | -1.763 | 0.087 |
|  | Goat | 0.306 | 0.816 | 34 | -1.352 | 1.964 | 0.375 | 0.71 |
|  | Impala | -0.079 | 0.395 | 34 | -0.882 | 0.724 | -0.2 | 0.842 |
|  | Sheep | -1.071 | 0.737 | 34 | -2.569 | 0.427 | -1.453 | 0.155 |
