# Supplementary material for: A cross-sectional study reveals complex associations between gastrointestinal parasite composition and faecal immune and inflammatory biomarkers in wildlife and livestock in Kenya
Source: Front Immunol. 2026 Jul 28;17:1881798. doi: 10.3389/fimmu.2026.1881798 (PMC13457056; doi:10.3389/fimmu.2026.1881798)
Supplement: Supplementary file 3 [file DataSheet3.docx]

**Supplementary Materials 3: Biomarker storage stability**

To test the stability of each biomarker in stored samples, we repeated assays on samples one year apart. We tested faecal extracts that had been stored at -20 °C, and re-extracted from faecal samples stored at -80 °C.

**Supplementary Table 3.1**: Pearson’s correlations between log10 transformed concentrations of each biomarker in samples run in 2024 and the same samples in 2025. New extracts were extracted form samples stored at -80°C, whereas old extracts were stored at -20°C.

**Supplementary Figure 3.1: Sample concentrations of each biomarker were significantly correlated between assays run one year apart**. We re-extracted faecal samples stored at -80 °C (blue), and re-ran extracts stored at -20 °C.
