# Supplementary material for: A cross-sectional study reveals complex associations between gastrointestinal parasite composition and faecal immune and inflammatory biomarkers in wildlife and livestock in Kenya
Source: Front Immunol. 2026 Jul 28;17:1881798. doi: 10.3389/fimmu.2026.1881798 (PMC13457056; doi:10.3389/fimmu.2026.1881798)
Supplement: Supplementary file 4 [file DataSheet4.docx]

**Supplementary Materials 4: Primer and Index Sequences**

*Nematode primer sequences*

ITS2 Forward - NC1: ACGTCTGGTTCAGGGTTGTT

ITS2 Reverse - NC2: TTAGTTTCTTTTCCTCCGCT

18S Forward - ecoF: GGTTAAAAMGYTCGTAGTTG

18S Reverse - ecoR: TGGTGGTGCCCTTCCGTCA

*Primer sequences with Illumina adapters*

NC1_0N: TCGTCGGCAGCGTCAGATGTGTATAAGAGACAGACGTCTGGTTCAGGGTTGTT

NC1_1N: TCGTCGGCAGCGTCAGATGTGTATAAGAGACAGNACGTCTGGTTCAGGGTTGTT

NC1_2N: TCGTCGGCAGCGTCAGATGTGTATAAGAGACAGNNACGTCTGGTTCAGGGTTGTT

NC1_3N: TCGTCGGCAGCGTCAGATGTGTATAAGAGACAGNNNACGTCTGGTTCAGGGTTGTT

NC2_0N: GTCTCGTGGGCTCGGAGATGTGTATAAGAGACAGTTAGTTTCTTTTCCTCCGCT

NC2_1N: GTCTCGTGGGCTCGGAGATGTGTATAAGAGACAGNTTAGTTTCTTTTCCTCCGCT

NC2_2N: GTCTCGTGGGCTCGGAGATGTGTATAAGAGACAGNNTTAGTTTCTTTTCCTCCGCT

NC2_3N: GTCTCGTGGGCTCGGAGATGTGTATAAGAGACAGNNNTTAGTTTCTTTTCCTCCGCT

ecoF:

TCGTCGGCAGCGTCAGATGTGTATAAGAGACAGGGTTAAAAMGYTCGTAGTTG

ecoR:

GTCTCGTGGGCTCGGAGATGTGTATAAGAGACAGTGGTGGTGCCCTTCCGTCA

*Index*

N501_i5 - TAGATCGC

N502_i5 - CTCTCTAT

N503_i5 - TATCCTCT

N504_i5 - AGAGTAGA

N505_i5 - GTAAGGAG

N506_i5 - ACTGCATA

N507_i5 - AAGGAGTA

N508_i5– CTAAGCCT

N510_i5 - CGTCTAAT

N511_i5 - TCTCTCCG

N513_i5 - TCGACTAG

N515_i5 - TTCTAGCT

N516_i5 - CCTAGAGT

N517_i5 - GCGTAAGA

N518_i5 - CTATTAAG

N520_i5 - AAGGCTAT

N701_i7 - TCGCCTTA

N702_i7 - CTAGTACG

N703_i7 - TTCTGCCT

N704_i7 - GCTCAGGA

N705_i7 - AGGAGTCC

N706_i7 - CATGCCTA

N707_i7 - GTAGAGAG

N708_i7 - CCTCTCTG

N709_i7 - AGCGTAGC

N710_i7 - CAGCCTCG

N711_i7 - TGCCTCTT

N712_i7– TCCTCTAC

N714_i7 - TCATGAGC

N715_i7 - CCTGAGAT

N716_i7 - TAGCGAGT

N718_i7 - GTAGCTCC

N719_i7 - TACTACGC

N720_i7 - AGGCTCCG

N721_i7 - GCAGCGTA

N722_i7 - CTGCGCAT

N723_i7 - GAGCGCTA

N724_i7 - CGCTCAGT

N726_i7 - GTCTTAGG

N727_i7 - ACTGATCG

*Full index sequences*

N501_i5 - AATGATACGGCGACCACCGAGATCTACACTAGATCGCTCGTCGGCAGCGTC

N502_i5 - AATGATACGGCGACCACCGAGATCTACACCTCTCTATTCGTCGGCAGCGTC

N503_i5 - AATGATACGGCGACCACCGAGATCTACACTATCCTCTTCGTCGGCAGCGTC

N504_i5 - AATGATACGGCGACCACCGAGATCTACACAGAGTAGATCGTCGGCAGCGTC

N505_i5 - AATGATACGGCGACCACCGAGATCTACACGTAAGGAGTCGTCGGCAGCGTC

N506_i5 - AATGATACGGCGACCACCGAGATCTACACACTGCATATCGTCGGCAGCGTC

N507_i5 - AATGATACGGCGACCACCGAGATCTACACAAGGAGTATCGTCGGCAGCGTC

N508_i5 - AATGATACGGCGACCACCGAGATCTACACCTAAGCCTTCGTCGGCAGCGTC

N701_i7 - CAAGCAGAAGACGGCATACGAGATTCGCCTTAGTCTCGTGGGCTCGG

N702_i7 - CAAGCAGAAGACGGCATACGAGATCTAGTACGGTCTCGTGGGCTCGG

N703_i7 - CAAGCAGAAGACGGCATACGAGATTTCTGCCTGTCTCGTGGGCTCGG

N704_i7 - CAAGCAGAAGACGGCATACGAGATGCTCAGGAGTCTCGTGGGCTCGG

N705_i7 - CAAGCAGAAGACGGCATACGAGATAGGAGTCCGTCTCGTGGGCTCGG

N706_i7 - CAAGCAGAAGACGGCATACGAGATCATGCCTAGTCTCGTGGGCTCGG

N707_i7 - CAAGCAGAAGACGGCATACGAGATGTAGAGAGGTCTCGTGGGCTCGG

N708_i7 - CAAGCAGAAGACGGCATACGAGATCCTCTCTGGTCTCGTGGGCTCGG

N709_i7 - CAAGCAGAAGACGGCATACGAGATAGCGTAGCGTCTCGTGGGCTCGG

N710_i7 - CAAGCAGAAGACGGCATACGAGATCAGCCTCGGTCTCGTGGGCTCGG

N711_i7 - CAAGCAGAAGACGGCATACGAGATTGCCTCTTGTCTCGTGGGCTCGG

N712_i7 – CAAGCAGAAGACGGCATACGAGATTCCTCTACGTCTCGTGGGCTCGG
